# Phase-Field Simulation on the Effect of Second-Phase Particles on Abnormal Growth of Goss Grains in Fe-3%Si Steels

**DOI:** 10.3390/nano12234148

**Published:** 2022-11-23

**Authors:** Mingtao Wang, Yongkai Xu, Jinlong Hu, Feng Fang, Jianfeng Jin, Tao Jia, Qing Peng

**Affiliations:** 1 School of Materials Science and Engineering, Northeastern University, Shenyang 110819, China; 2 State Key Laboratory of Rolling and Automation, Northeastern University, Shenyang 110819, China; 3 State Key Laboratory of Nonlinear Mechanics, Institute of Mechanics, Chinese Academy of Sciences, Beijing 100190, China

**Keywords:** phase-field simulation, abnormal grain growth, Fe-3%Si steel, second-phase particle, Goss grain

## Abstract

A phase-field model was revised to study the abnormal growth of Goss grains during the annealing process in Fe-3%Si steels, in which the interaction between the second-phase particles and Goss grain boundaries (GBs) was considered. The results indicate that the abnormal growth of Goss grains occurs due to the different dissolvability of the particles at Goss GBs compared with the other GBs. Moreover, the degree of abnormal growth increases first and then decreases with an increasing particle content. Meanwhile, the size advantage of Goss grain can further promote the degree of abnormal growth. Two types of island grains were found according to the simulated results, which is consistent with the experimental observations. A proper GB dissolvability of particles is the key factor for the formation of isolated island grains, and a higher local particle density at GBs is the main reason for the appearance of serial island grains. These findings can provide guidance for the desired texture control in silicon steels.

## 1. Introduction

Abnormal grain growth during recrystallization, namely, when a few grains consume their surrounding grains and thus experience a rapid growth process after the completion of the initial recrystallization, has been applied in silicon steel manufacture for many years [1]. In silicon steels, the control of Goss grains’ abnormal growth is the key to the desired texture, especially in the presence of second-phase particles. The mechanism of abnormal growth has been extensively studied, such as the size dominance mechanism by May and Turnbull [2], the aggregation nucleation theory proposed by Inokuti [3], and the solid-state wetting theory proposed by Hwang [4]. The above theories have either been disproved or not verified by experiments [5,6,7]. As concerning the influence of GB characteristics and second-phase particles on abnormal growth, there are two widely accepted theories at present, namely, coincident site lattice theory (CSL) proposed by Harase [8,9] and high energy grain boundary theory (HE) proposed by Hayakawa [10]. The main point in common of the two theories is that some special GB types are easily formed around Goss grains (such as ∑3–9 in the CSL model and 15–45° disorientation in HE model), and the particles can easily dissolve at that particular types of GBs, resulting in a reduction or even disappearance of the pinning effect. Then some Goss grains show a high expanding ability compared with other grains, and abnormal growth could be caused eventually. Thus, it could be speculated that there is a close relationship between the abnormal growth of Goss grains and the special behavior of particles at the GBs during the process. The mechanism was investigated by many researchers from different perspectives by means of experiments; however, it is difficult to directly observe the entire abnormal growth process and quantitatively describe the complex interaction between particles and GBs [11,12,13].

The phase-field method is an effective computational simulation method for characterizing the microstructure evolution and has been used in many areas [14,15,16,17,18,19,20,21]. Many phase-field models have been developed for grain growth in a multi-grains system [22,23,24,25]. Meanwhile, some models were developed to simulate the evolution of the microstructure with second-phase particles. The particles, expressed by a new field variable, were introduced in a multi-grain system based on the modification of local free energy by Moelans [26]. Kunok et al. accessed the influence of particles characteristics such as shape, distribution, and size on the microstructure morphology in 2D and 3D [27,28,29,30]. He et al. quantitatively analyzed the microstructure evolution affected by the content and size of particles in the AZ31 alloy [31]. Ashise et al. developed a phase-field model for grain growth in the presence of mobile particles driven by the GB curvature [32,33]. The hindering effect of particles on the GB is mainly considered in most models; however, there is no model for the complex relationship between the effect of particles on GBs with varying content due to dissolution, as is the case in some silicon steels. In addition, most particles, such as MnS and AlN, in Fe-3%Si alloys are nanoscale, while the Goss grains undergoing abnormal growth are microscale [8,9,10]. The huge size difference makes it difficult to simulate the interaction between the particles and grains directly.

In this study, a phase-field model including the particle pinning item was revised in Fe-3%Si steel, a typical silicon steel grade, under the real temporal and spatial conditions on an industrial scale. The expression of local free energy function coupled with the pinning effect item with a varying particle content was proposed based on Zener pinning theory. The complex interaction between second-phase particles and Goss GBs was quantitatively studied to explore their effects on abnormal growth. Finally, the mechanism of abnormal growth in the Fe-3%Si steel system was clarified, and it is of great significance in material design and process optimization. This model efficiently explores the interaction between nanoparticles and GBs on a mesoscopic scale using the equivalent pinning force based on Zener theory. Meanwhile, the modeling performed in this work can also be utilized for the understanding of the physics of nanocrystalline alloys, which are driven by the same mechanism.

## 2. Materials, Model, and Parameters

### 2.1. Phase Model with Second-Phase Particles

The phase-field model is a versatile mathematical tool for studying boundary evolution in materials. Its flexibility makes it powerful in tracking the microstructure evolution of a dynamic system. In this work, a phase-field model considering the dissolvable particles was developed based on the model, which has been successfully applied for describing the grain growth process [23,24]. Here, with the value determination rules proposed by our team [34], the simulation of microstructure evolution in Fe-3%Si steels with second-phase particles was performed.

Normally, the typical microstructure of Fe-3%Si steels with particles is featured with polycrystalline ferrite grains and dispersed particles, such as MnS and AlN [35,36]. Thus, a set of variables related to orientations was chosen for the characterization of different grains. For a polycrystalline system with *n* grains, a long-range order parameter *η_q_* is defined for the *q*-th grain. Each *η* has a value between 1 and 0, 1 for the grain interior, 0 for outside of grain, and between 0 and 1 for the GB area. A constraint for the phase-field values is that the sum of these in a grid point should be 1, $\sum_{q=1}^{n}\eta_{q}^{2}\left(r,t\right)=$ 1. All variables can be obtained by solving the time-dependent Ginzburg–Laudau equation [37] as follows:

(1) $$\;\;\frac{\partial \eta_{p}\left(r,t\right)}{\partial t}=-L\frac{\delta F}{\delta \eta_{p}\left(r,t\right)},\;p=1,2,3\ldots n\;$$

where *t* is time, ***r*** is position, *p* is orientation index, *L* is a variable related to grain coarsen mobility, and *F,* as the function of free energy, can be expressed as follows in the system:

(2) $$F=\int \left[f_{0}\left(\eta_{1}\left(r,t\right),\eta_{2}\left(r,t\right),\ldots ,\eta_{n}\left(r,t\right)\right)+\frac{K_{2}}{2}\sum_{q=1}^{n}{\left(\nabla \eta_{q}\left(r,t\right)\right)}^{2}\right]dr\;$$

(3) $$\;f_{0}=A+\frac{B_{1}}{2}\sum_{q=1}^{n}\eta_{q}^{2}\left(r,t\right)+\frac{B_{2}}{4}\sum_{q=1}^{n}\eta_{q}^{4}\left(r,t\right)+\frac{K_{1}}{2}\sum_{q=1}^{n}\sum_{p\neq q}^{n}\eta_{q}^{2}\left(r,t\right)\eta_{p}^{2}\left(r,t\right)+P_{Z}$$

In Equation (2), *f_0_* is local free energy related to thermodynamic properties and stored energy during the deformation [31]. $\frac{B_{1}}{2}\sum_{q=1}^{n}\eta_{q}^{2}\left(r,t\right)+\frac{B_{2}}{4}\sum_{q=1}^{n}\eta_{q}^{4}\left(r,t\right)$ represents a multi-well potential with minimal value of zero based on the relationship of *B*_1_ = *B*_2_. Specifically, *B*_1_ and *B*_2_ are coefficients related to cold deformation stored energy inside the grains, which can be calculated by *f_stored energy_* = *f*_0_(∑*η*^2^ = 0) − *f*_0_(∑*η*^2^ = 1). *K*_1_ and *K*_2_ are related to the GB and its range, since they only work in the GB area due to *η* varies through the GB and two *ηs* with non-zero values in one grid exits. In this study, different pinning effects of particles on Goss GBs and other GBs during the grain growth in the steels is desired. More details on the model can be found in Refs. [23,24,26]. In order to describe the behaviors in different sites of particles, a pinning force item *P_z_* was introduced in the model as follows:

(4) $$\;P_{Z}=S\left(r,\chi \right)\left(D\left(r,\varepsilon \right){|}_{S\left(r,\chi \right)=1}\right)\sum_{p=1}^{n}\eta_{p}^{2}$$

where *S*(***r***,χ) indicates the position of particles and is given as follows:

(5) $$S\left(r,\chi \right)=\frac{1}{\chi -\mu }\langle \chi -\mu \;,\;0\rangle$$

where <*χ*-*μ*,0> = *max*(*χ*-*μ*,0), χ is a random number in [0, 1], *μ* is a threshold value related to the particle content and size, and different *μ*s correspond to a changing content of particles. *D*(***r***,*ε*)|_*S*(***r***,*χ*) = 1_ means the dissolving probability of particles at GBs due to the coarsening of particles in their vicinity.

(6) $$D\left(r,\varepsilon \right){|}_{S\left(r,\chi \right)=1}=\frac{\sigma }{\varepsilon -\omega }\langle \varepsilon -\omega \;,\;0\rangle$$

where <*Ɛ*-*ω*,0> = *max*(*Ɛ*-*ω*,0), σ is a coefficient related to the pinning force level, *Ɛ* is a random number in [0, 1], *ω* is a threshold value related to the dissolvability and different *ω*s correspond to varying dissolvability of particles. It should be noted that this term only works at the Goss GBs, which is achieved by assigning the *ω* = 0 to the GBs between non-Goss grains. Finally, the particle content and the dissolution of the Goss GBs to particles can be obtained by different threshold values of *μ* and *ω*. In short, $S\left(r,\chi \right)\left(D\left(r,\varepsilon \right){|}_{S\left(r,\chi \right)=1}\right)$ was introduced into this model to quantitatively describe the content and dissolution of particles. According to Equation (4), a pining force caused by a particle exists in the position when $S\left(r,\chi \right)\left(D\left(r,\varepsilon \right){|}_{S\left(r,\chi \right)=1}\right)=\sigma$, and *P_z_* is non-zero so a pinning effect contributes to the coarsening process. Otherwise, the grid point is not in the range of pining force $S\left(r,\chi \right)\left(D\left(r,\varepsilon \right){|}_{S\left(r,\chi \right)=1}\right)=0$ and *P_z_* does not serve any function. More details are shown as a flow chart in 2.2.

### 2.2. Model Parameters Setting and Initial Condition

To accomplish the simulation under the real temporal and spatial conditions, all the parameters in the model should be taken based on the physical definition. Kinetic data on normal recrystallization coarsening in the Fe-3%Si system without particles is necessary for parameter calibration and model verification [31]. The chemical composition of the Fe-3%Si steel without particles is *ω*(Si) = 3.26%, *ω*(C) = 0.004% with rest of Fe. The steel production process included strip casting [38,39], cold rolling with a reduction of 83.3%, and a following annealing. In this research, the grain coarsening during the annealing process was simulated at the annealing temperatures between 1123 K and 1323 K.

Considering the limited computing resource, we implemented the simulation in a 2D system with the APT algorithmic strategy [40] for the arbitrary orientations of grains. The values of *B*_1_ and *B*_2_ are equal and related to the stored energy fully released during annealing and are independent of temperature. The values were taken as 51.2 J/mol in this model according to Ref. [34]. The local free energy inside the grain can be determined based on THERMOCALC data with the defined composition and temperature. The parameter *A* can be obtained based on Equation (3) with $f_{0}\left(\eta_{p}^{2}=1,\sum_{q\neq p}^{n}\eta_{q}^{2}=0\right)$ and the values are −71.21 KJ/mol (1123 K), −78.48 KJ/mol (1223 K), and −85.83 KJ/mol (1323 K), respectively. The GB scope in phase-field model is not the exact physical size [34] and the range was taken as 1.80 μm (3 grids) in this simulation. Based on the model analysis, the GB range is mainly decided only by *K_2_* [34], which was taken as 9.21 × 10^−12^ J∙m^2^/mol. The large angle GB energy was set as 0.55 J/m^2^ according to its normal range of 0.5–0.6 J/m^2^ in a polycrystalline system [5]. Meanwhile, the GB energy can be expressed as a function of *K*_1_ and *K*_2_ [41], then *K*_1_ can be determined as 81.92 J/mol based on determined *K*_2_.

**Figure 1:**
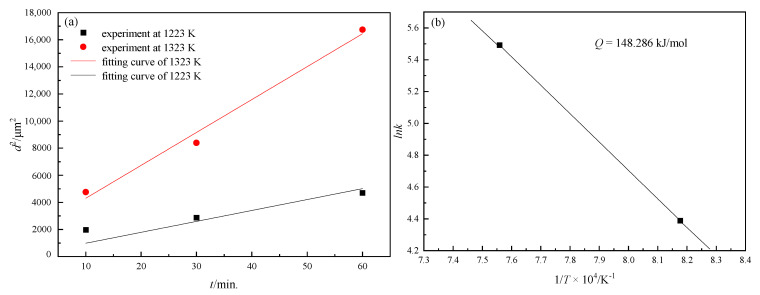
(**a**) Grain growth curve of Fe-3% Si steel at 1223 and 1323 K, (**b**) relationship between *lnk* and *1/T*.

According to Ref. [34], *L* in Equation (1), related to the GB mobility, is a function of temperature and can be expressed as follows:

(7) $$L=L_{0}e^{-\frac{Q}{RT}}$$

where *R* is the gas constant, *Q* is grain growth activity energy in this simulation and *L*_0_ is a constant depending on the alloy system. A series of heat treatment tests on the Fe-3%Si steel (without the second-phase particles) were implemented for the determination of *Q.* Grain size increases with annealing time and the coarsening rate increases at a higher temperature, which agrees well with the following relationship (Figure 1a):

(8) $$d^{2}-d_{0}^{2}=kt$$

where $k=a\times e^{-\frac{Q}{RT}}$, and *Q* can be determined by −RTln(k/a) as seen in Figure 1b.

**Figure 2:**
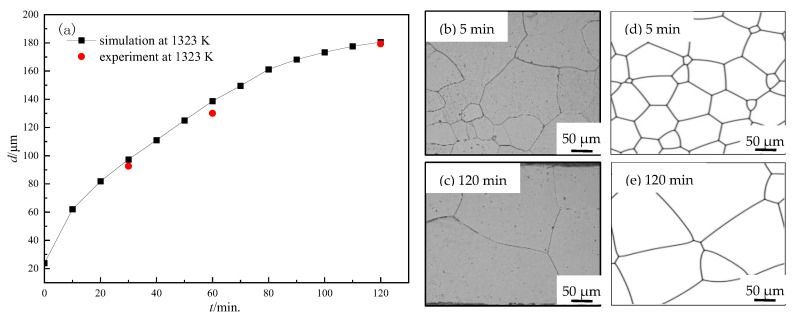
Comparison of simulated and experimental results for the Fe-3%Si steel at 1323 K. (**a**) Average grain size, (**b**,**c**) microstructures of testing samples at 5 min and 120 min, (**d**,**e**) simulated microstructures at 5 min and 120 min.

*L* cannot be derived directly, and we calibrated it by the comparison of simulated and experimental data at a temperature, which was chosen as 1323 K in this simulation. It is found that the grain growth kinetic curve from simulation data fits (see Figure 2) the experimental results well when *L* = 3.97 mol/(J∙s) at 1323 K. The simulated results are compared with experimental data, as shown in Figure 2. Thus, *L*_0_ can be determined as 2.84 × 10^6^ m^3^s^−1^J^−1^, and *L* at arbitrary temperature can be calculated based on Equation (7).

**Figure 3:**
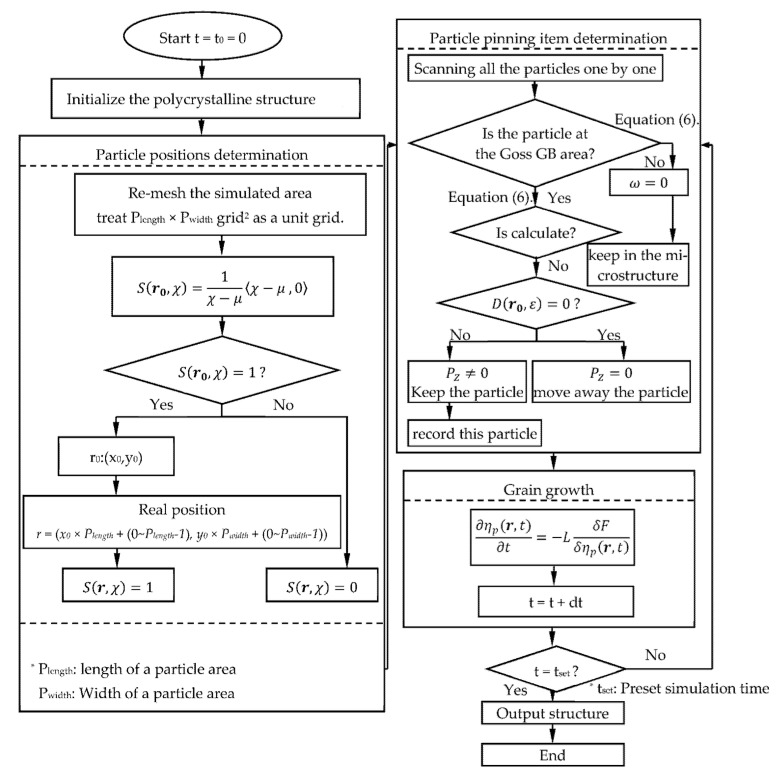
Flow diagrams of phase-field model in this work. The polycrystalline structure initialization refers to [34].

**Figure 4:**
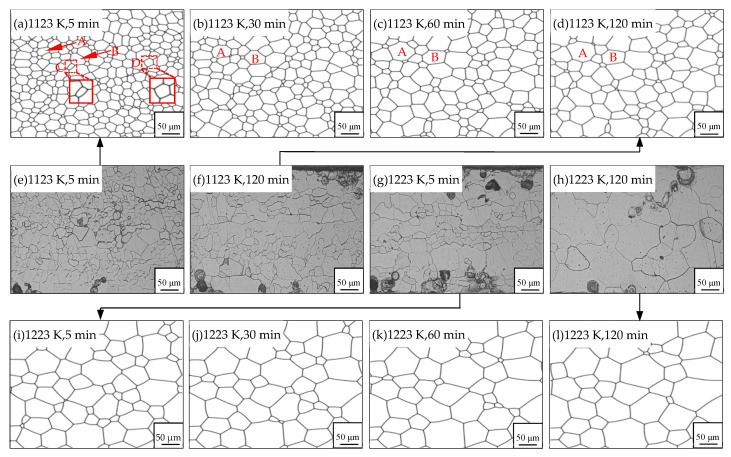
The coarsening process of simulated data and experimental results at 1123 K and 1223 K for 120 min: (**a**–**d**), (**i**–**l**), simulated microstructures; (**e**–**h**): experimental observations.

In order to further clarify the idea of the model, especially the treatment of the second-phase particles, a flow diagram for describing the model was applied as follows (Figure 3):

Based on the model and parameters, the program written with FORTRAN was run on a server cluster. Equation (1) was solved by an explicit finite difference method in 2D with a periodic boundary condition. Square grids with a size of 0.6 μm × 0.6 μm were adopted and 600 × 800 (for microstructure comparison in Figure 4), 512 × 512 (for single Goss grain evolution), and 2048 × 4096 (for Goss grains with varying ωs evolution) grid divisions were applied for the simulations.

Above all, a phase-field model for the description of the microstructure evolution with changing particle contents in Fe-3%Si was utilized. All parameters were confirmed uniquely through theoretical calculation and experimental data calibration. Utilizing this phase-field model, the interaction between the second-phase particles and Goss GBs can be studied quantitatively.

## 3. Result and Analysis

### 3.1. Grain Growth in Non-Oriented Fe-3%Si Steels at Different Temperatures

In this study, the microstructure evolution of non-oriented Fe-3%Si steels without inhibitors was simulated at the annealing temperatures of 1123 K and 1223 K. Meanwhile, the comparison between experimental data and simulated results was made for the model testing, as can be seen in Figure 4. Both the simulated results and experimental observations follow the same coarsening process. It is clear that a normal coarsening of grains driven by the GB curvature can be seen. The gradual flattening of GBs reduces the GB energy of the system, resulting in the phenomenon of the smaller grain being consumed by the larger one.

**Figure 5:**
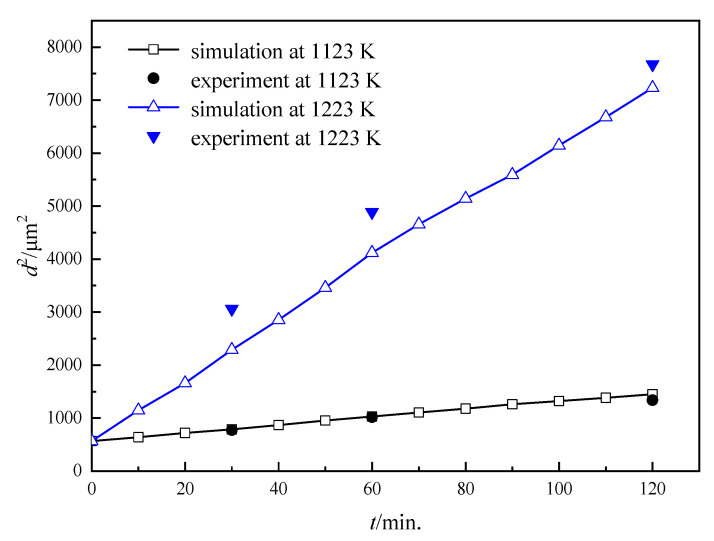
Comparison between simulated data and experimental results at 1123 K and 1223 K.

Furthermore, a quantitative analysis of the microstructure evolution is shown in Figure 5. It can be known that in the non-oriented Fe-3%Si steel, average grain area (d^2^) vs. time follows a linear relationship during coarsening, which means the process is mainly controlled by the GB curvature. Based on the data comparison, the maximum difference in average grain size between the experimental and simulated data happens at 1223 K, 60 min, and the error value is 8.7%, which means a good match between the simulated and experimental results. Thus, the correctness and validation of this model and suitable parameters can be demonstrated.

Further investigation on the grain area variation reveals that the relationship between the growth rate of individual grains and the grain topological level *n* (the number of sides of a grain) in a 2D model, proposed by von Neuman and Mullins based on grain growth kinetics [42], still works in the simulated system.

(9) $$\frac{dA}{dt}=\frac{\pi M\gamma }{3}\left(n-6\right)$$

where *A* is the grain area, *M* is the mobility, γ is GB energy. According to Equation (9), grains with less than 6 sides have a negative derivative of the area with respect to time, which means the grains could shrink and vanish. While grains with more than 6 sides have a positive derivative of the area with respect to time, so their area continues to increase as the microstructure evolves. This behavior can be easily captured by this model as shown in Figure 4. It can be seen that grain A with 7 sides and grain B with 9 sides both undergo coarsening, however, grain C with 4 sides and grain D with 5 sides shrink and disappear subsequently.

### 3.2. Influence of the Particles on the Grain Growth Process

The reduction of GB energy is the main driving force for grain growth. For an ideal microstructure, the abnormal growth is unlikely to occur in the absence of impurities under a fixed GB energy as discussed in 3.1. The second-phase particles, also known as inhibitors, such as AlN and MnS, have been introduced into silicon steels to trigger the abnormal growth in the industry [43,44,45]. Particles diffusely distribute in the microstructure and contribute to the pinning effects on GB movement during grain growth. Based on Zener pinning theory derived in the model with GBs and randomly distributed particles, the pinning force on unit GB can be expressed as follow [46]:

(10) $$F_{Z}=\frac{3\sigma_{gb}f_{v}}{4r}$$

where *f_v_*, *r*, and *σ*_gb_ are the particle content, size, and GB energy, respectively. It can be speculated that the pinning force mainly depends on the ratio of the content to the size of particles according to Equation (10). In our model, the grid was taken as 0.6 μm, considering the computational efficiency and the appropriate GB scope. Normally the particle content in the microstructure is less than 0.2% and the average size is 20 nm~30 nm [47], which is much smaller than the simulation grids. Thus, a round area with a size of 2 grids was chosen as the particle pinning site, and the content was taken as 0~8% for the equivalent pinning effect in a real alloy based on Equation (10). The conversion relation between the real particle content in Fe-3%Si steels and the particle pinning site content (1-μ) in the model can be expressed as *c* = (1-μ) × *r*_particle in alloys_/*r*_particle in model_. Then the corresponding particle content c can be taken as 0~0.20%, corresponding to a μ of 1~0.92 based on a real particle size of 30 nm.

In the model as shown in Equation (6), the threshold value ω indicates the particle dissolvability of GBs, ω = 1 means all the particles dissolve, and ω = 0 means all the particles are stable. In this work, the ω of non-Goss grains was taken as 0, representing all the particles at the non-Goss GBs are invariable. The effect of Goss GBs with different particle dissolvabilities on the microstructure evolution process can be explored by changing values of ω at Goss GBs. The coefficient σ in Equation (6) was taken as 1 for simplification. In this study, the simulation was carried out at 1273 K with a maximum holding time of 100 min. A Goss grain with an average size was placed in the central region of the initial microstructure.

**Figure 6:**
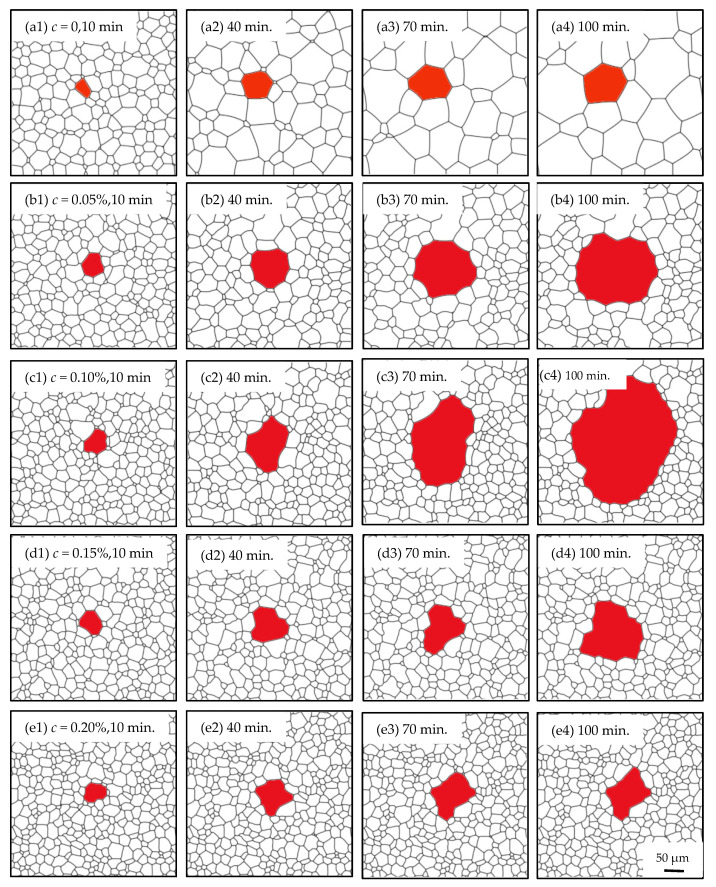
Microstructure evolution under different contents (0~0.20%) where *T* = 1273 and *ω* = 0.90. (**a_1_**–**a_4_**) *c* = 0, 10–100 min; (**b_1_**–**b_4_**) *c* = 0.05%, 10–100 min; (**c_1_**–**c_4_**) *c* = 0.10%, 10–100 min; (**d_1_**–**d_4_**) *c* = 0.15%, 10–100 min; (**e_1_**–**e_4_**) *c* = 0.20%, 10–100 min.

In order to investigate the effect of the particle content on the microstructure evolution characteristics, ω for the Goss grain was taken as 0.90 in the simulation. The morphology during the 100-min annealing is shown in Figure 6. It can be seen that, with the increasing of c, the average grain size of non-Goss grains decreases significantly, and the abnormal growth level increases first and then decreases, reaching a maximum at *c* = 0.10%. It is also found that the normal growth of the non-Goss grains is significantly inhibited and even stalled with *c* = 0.15%, 0.20%, and a significant abnormal growth occurs. Meanwhile, as *c* = 0.05%, the Goss grain has a certain size advantage over the non-Goss grains, although the degree of abnormal growth is slighter. When *c* = 0, there are no particles in the microstructure, and abnormal growth does not happen. In this case, the normal growth of non-Goss grains cannot be suppressed, and the Goss grain has the same behavior as non-Goss ones.

**Figure 7:**
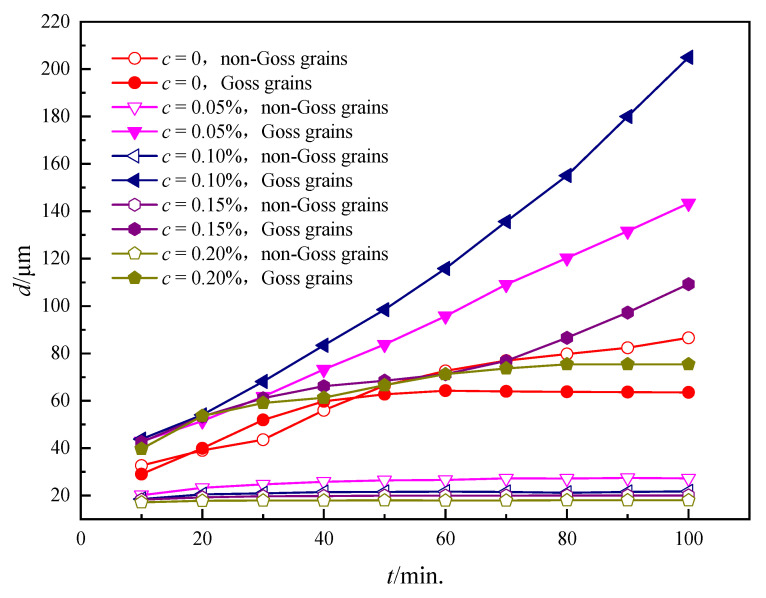
The kinetic curves for Goss and non-Goss grains under different particle contents where *T* = 1273 K and *ω* = 0.90.

Figure 7 shows the kinetic curves of grain growth under different *c*. It can be seen that, under a fixed particle content (except *c* = 0), the average size of non-Goss grains increases slightly, however, the size of Goss ones increases a lot. When *c* = 0, Goss grain and non-Goss grains follow a similar kinetic behavior. At the same annealing time, the size of Goss grains goes through a maximum at *c* = 0.10%, following the same trend as the microstructure evolution featured in Figure 6.

**Figure 8:**
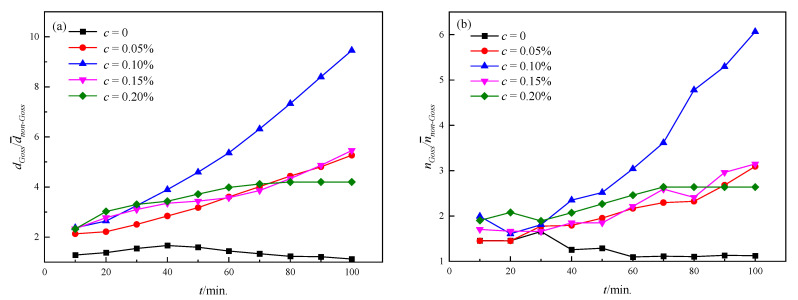
The quantitative characterization of abnormal growth levels under different particle contents: (**a**) the size ratio of Goss grains to non-Goss grains; (**b**) the side ratio of Goss grains to non-Goss grains.

In order to quantitatively characterize the level of abnormal growth, the size ratio of Goss grains to non-Goss grains has been calculated, as shown in Figure 8a. It is obvious that the ratio follows the same trend as the microstructure observation in Figure 6, and the ratio increases with the microstructure evolution (except *c* = 0). In order to further explore the mechanism of such a phenomenon, the change of the side ratio of Goss grains to non-Goss grains ($n_{Goss}/{\bar{n}}_{non-Goss}$) with time for different contents has been calculated as shown in Figure 8b. It is found that the ratio increases with annealing time except *c* = 0, which also agrees well with the size ratio.

The simulated results in Figure 6, Figure 7 and Figure 8 indicate that the particles contribute to the abnormal growth. It can be illustrated from the following two aspects: on the one hand, the higher the particle content means the stronger the pinning force on GBs based on Zener theory; on the other hand, the particle dissolvability of Goss GBs reduces the hindering effect on Goss GBs to a certain extent. Therefore, when *c* = 0, there is no pinning effect on either Goss grains or non-Goss grains, and they both follow a similar growth kinetic. When *c* > 0, with a low particle content, both non-Goss grains and Goss grains are subject to a weaker pinning, and Goss grains have a slight growth advantage compared to non-Goss ones; with a high particle content, both grains could be hindered by more particles, and the further grain growth would be different. Finally, the most obvious abnormal growth can be induced with a proper *c,* which is 0.10% in this simulation. According to Equation (9), the grain growth rate is proportional to the number of grain sides under the same GB energy, which means the grain size (usually larger grains have more sides) advantage itself could also promote the abnormal growth. Although the size dominance theory has been disproved by numerous experiments [5,6,7,48], which refers to the fact that the abnormal growth is not due to the initial size dominance of Goss grains, the large size (means large $n_{Goss}/{\bar{n}}_{non-Goss}$) itself is a factor promoting further abnormal growth once the Goss grains undergo rapid growth.

**Figure 9:**
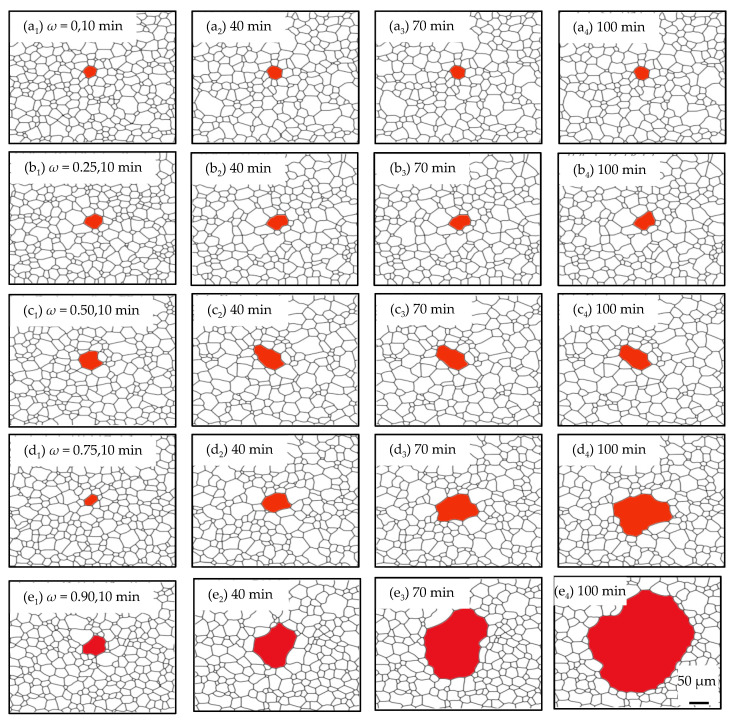
Microstructure evolution under various *ω*s (0–0.90) where *T* = 1273 K and *c* = 0.10%. (**a_1_**–**a_4_**) *ω* =0, 10–100 min; (**b_1_**–**b_4_**) *ω* = 0.25, 10–100 min; (**c_1_**–**c_4_**) *ω* = 0.50, 10–100 min; (**d_1_**–**d_4_**) *ω* = 0.75, 10–100 min; (**e_1_**–**e_4_**) *ω* = 0.90, 10–100 min.

In order to assess the influence of various particle dissolvabilities of Goss GBs on the coarsening process, the study on the microstructure evolution was carried out under different *ω*s from 0 to 0.90 and a constant particle content (*c* = 0.10%). In Figure 9, it can be seen that Goss grains expand with the consumption of their adjacent grains, which are pinned by the particles during the annealing process. Goss grains with various *ω*s show different growth rates and thus exhibit different abnormal growth levels. Specifically, abnormal growth level increases with an increasing *ω.*

**Figure 10:**
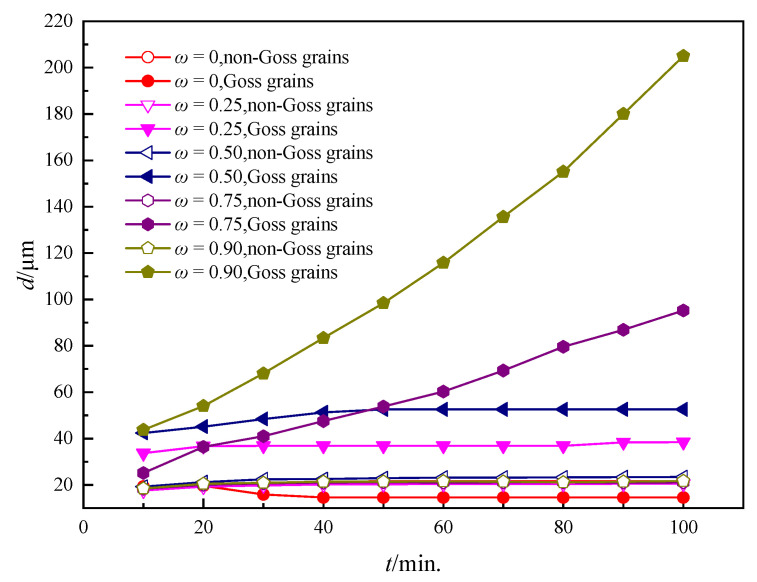
The kinetic curves of growth for Goss and non-Goss grains under different *ω*s where *T* = 1273 K and *c* = 0.10%.

The grain growth kinetic curves under different *ω*s are shown in Figure 10, which agrees well with the microstructure observations. It can be seen that the non-Goss grains have a similar average grain size due to the same particle content. With an increasing *ω*, the grain size difference between Goss grains and non-Goss grains becomes larger and larger, causing a higher abnormal growth level.

**Figure 11:**
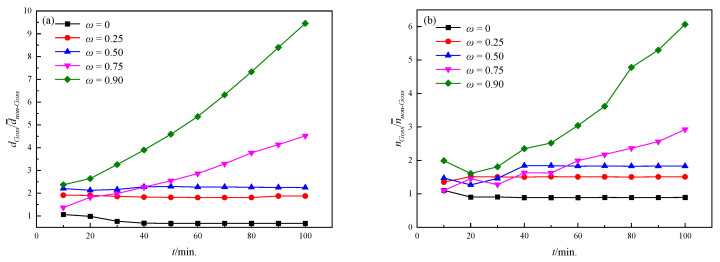
The quantitative characterization of abnormal growth levels under different *ω*s: (**a**) the size ratio of Goss grains to non-Goss grains; (**b**) the side ratio of Goss grains to non-Goss grains.

In order to quantitatively characterize the level of abnormal growth, the size ratio of Goss grains to non-Goss grains was calculated as shown in Figure 11a. It is obvious that the ratio increases with an increasing ω in the same annealing time and the ratio also increases during coarsening under a fixed ω. In order to further quantitatively clarify the mechanism, the change of the ratio of Goss grain sides to non-Goss ones with time under different ωs is shown in Figure 11b. It is found that the side ratio increases significantly with annealing time when ω is larger than 0.75, resulting in faster growth and causing abnormal growth behavior; when ω is lower than 0.75, the number of Goss grain sides remained the same or even decreased due to the accelerated coarsening of the non-Goss grains. The simulated results indicate that abnormal growth can occur when ω is larger than 0.75. It can be explained that, with the strengthening of the particle dissolvability of Goss GBs (an increasing ω), the pinning force on Goss grains is gradually weakened and abnormal growth becomes obvious under the same particle content.

**Figure 12:**
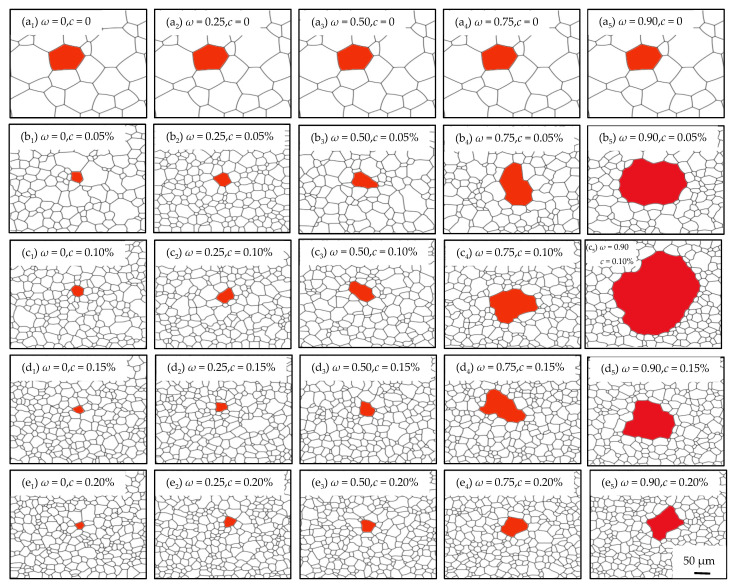
Microstructure evolution at 100 min under various combinations of *c* and *ωs* at 1273 K. (**a_1_**–**a_5_**) *c* = 0, *ω* = 0–0.90; (**b_1_**–**b_5_**) *c* = 0.05%, *ω* = 0–0.90; (**c_1_**–**c_5_**) *c* = 0.10%, *ω* = 0–0.90; (**d_1_**–**d_5_**) *c* = 0.15%, *ω* = 0–0.90; (**e_1_**–**e_5_**) *c* = 0.20%, *ω* = 0–0.90.

For the quantitatively comprehensive effects on microstructure evolution characteristics caused by c and ω, simulations were carried out in the cases of *c* = 0, 0.05%, 0.10%, 0.15%, 0.20%, and ω = 0, 0.25, 0.5, 0.75, 0.90. As shown in Figure 12, the size of non-Goss grains mainly depends on c, namely, the average grain size decreases with an increasing c. It also can be known that different combinations of c and ω result in different abnormal growth effects, in which b5, c5, and d5 exhibit the most obvious abnormal growth, while a1~a5, b1~b2, c1~c3, d1~d3, and e1~e2 only show normal grain growth.

**Figure 13:**
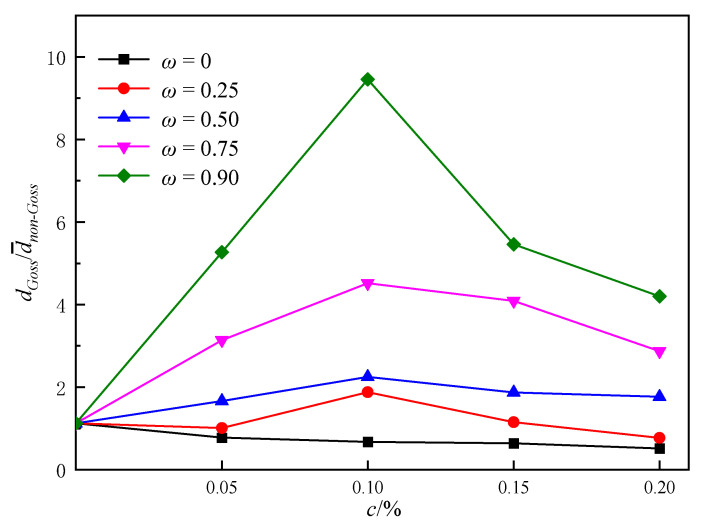
The size ratio of Goss grains to non-Goss grains under various *c* and *ω*s.

The size ratio of Goss grains to non-Goss gains at 100 min was analyzed as shown in Figure 13. It can be known that in the case of a constant *c*, the larger *ω* is, the higher the abnormal growth level is. The reason is that a larger *ω* means a higher dissolvability of Goss GBs, and thus the difference in mobility between the Goss grains and non-Goss ones becomes larger. Furthermore, with an increasing *c*, the mobility difference between Goss and non-Goss grains increases, and a higher level of abnormal growth can be found. While in the case of a constant *ω*, the situation is more complex. When *ω* = 0, which means there is no dissolvability on Goss GBs, Goss grains and non-Goss grains follow the same evolution process characterized by $d_{Goss}/{\bar{d}}_{non-Goss}$~1, and the average grain size decreases with an increasing particle content. When *ω* is large, such as 0.90, most particles dissolve when they meet with Goss grains, and the Goss grains tend to grow abnormally. In addition, more particles exit, the larger $d_{Goss}/{\bar{d}}_{non-Goss}$ shows with a maximum of 9.5 when *ω* = 0.90, *c* = 0.10%. When *ω* is between 0 and 0.90, $d_{Goss}/{\bar{d}}_{non-Goss}$ reaches the peak at a particle content of 0.10%, indicating that too high or too low particle content will prevent abnormal growth of Goss grains from occurrence when particles partially dissolve at Goss GBs. 

Thus, it can be known that abnormal growth can be induced by the addition of particles, and the level depends on the combined effect of the particle content and the dissolvability of Goss GBs. When the dissolvability is strong and the particle content is high, non-Goss grains are pinned by particles, and Goss grains exhibit fast evolution. This will result in a rapid increase in the number of Goss grain sides, which can further accelerate its growth and cause abnormal growth. In contrast, when the dissolvability is weak or the particle content is low, Goss grains are pinned and non-Goss grains are free to grow. There is a faint difference in mobility between the Goss grains and non-Goss ones, which results in low-level abnormal growth or even normal growth of the microstructure.

In order to further investigate the combined effects of Goss grains under different particle dissolvabilities, which are common in real microstructure evolution, the model was applied to study the evolution characteristics of the microstructure in the presence of grains with various *ω*s. In this study, *c* was taken at 0.10% as the initial condition for an apparent abnormal growth. The simulation was carried out for 140 min at 1273 K. A few grains under different *ω*s in the range of 0.30~0.98 were distributed randomly in the microstructure.

**Figure 14:**
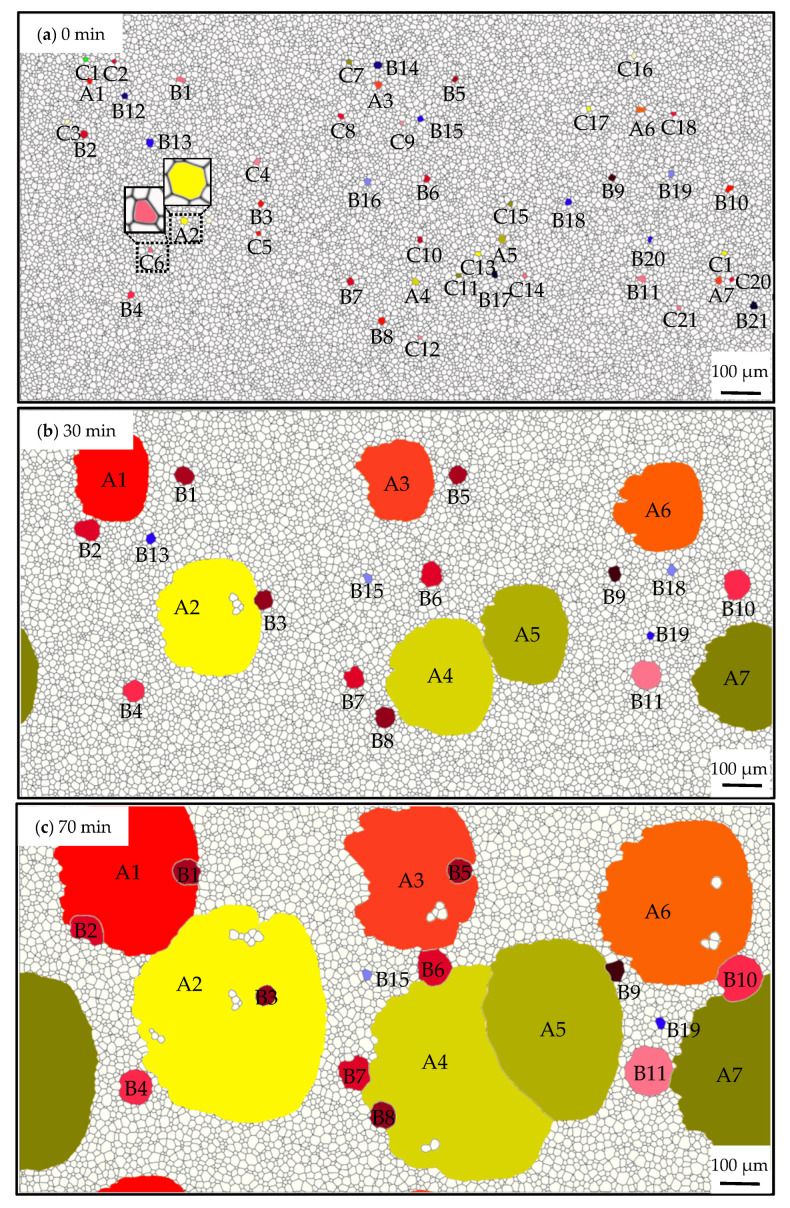

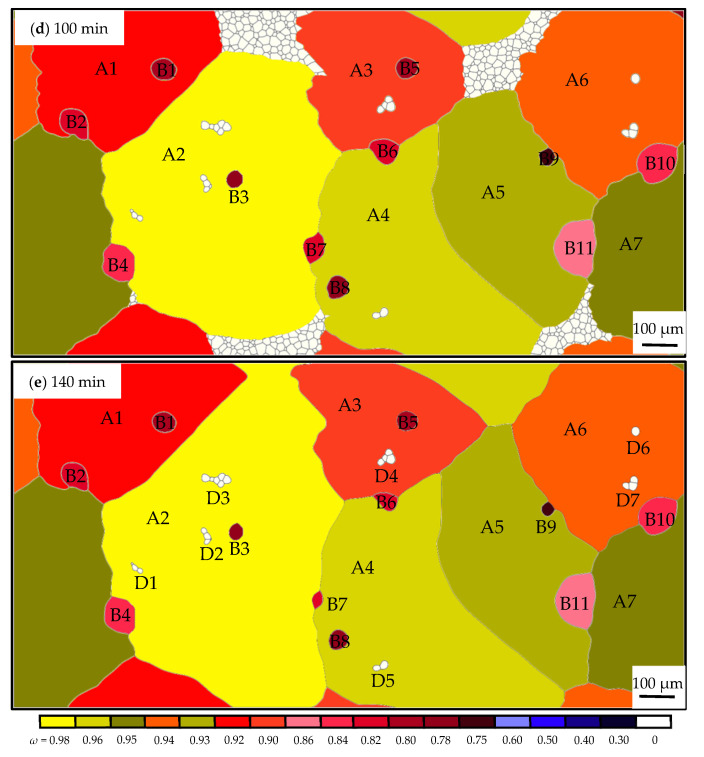
Simulated microstructure under various *ω*s and a fixed *c* of 0.10% at 1273 K within 140 min. (**a**–**e**) the microstructure evolution form 0~140 min. A1~A7: Goss grains with high *ω* of 0.98~0.90; B1~B21: other grains with medium *ω* of 0.86~0.30; C1~C21:grains with sides less than 6 and various *ω*s.

Figure 14a shows the initial microstructure, and grains with different *ω*s can be distinguished by their color. Goss grains with high *ω* are labeled with A1~A7 and some other grains with medium *ω* are B1~B21, meanwhile, these grains own not less than 6 sides and their *ω*s are 0.98~0.90 and 0.86~0.30, respectively. Grains labeled with C1~C21 refer to grains with sides less than 6 and various *ω*s. With the microstructure evolution, grains with sides more than 6 expand, and an obvious size advantage can be observed after 40 min (Figure 14b). A1~A7 develop into huge grains, and B1~B11 are also kept in the microstructure, as shown in Figure 14e. On the contrary, C1~C21 shrinks and disappears, indicating all the grain’s evolution is driven by the GB curvature. It also can be seen that the higher *ω*, the faster-coarsening rate, and the larger grain size is obtained. The weaker pinning effect caused by the particles contributes to the growth advantage of Goss grains, and the size of grains under a high *ω* becomes larger compared with those under a low *ω*. Then a larger grain size means more sides, which also leads to a greater growth speed. Therefore, it is speculated that the weaker pinning force and the consequential size advantage result in a greater advantage in growth and cause abnormal growth of Goss grains.

According to the simulated results, as shown in Figure 14, some island grains appeared during the process. There are two types of island grains in the microstructure, as shown in Figure 14, namely, B1~B11 and D1~D7. B1~B11 are grains with a medium *ω*, this kind of grain has a growth rate between Goss grains and normal grains with a *ω* = 0. Thus, the size of the grains is large enough and difficult to be engulfed by other grains. As to the normal grains inside the Goss grains, the reason mainly relies on the pinning effect caused by the particles, which did not dissolve at the GBs.

**Figure 15:**
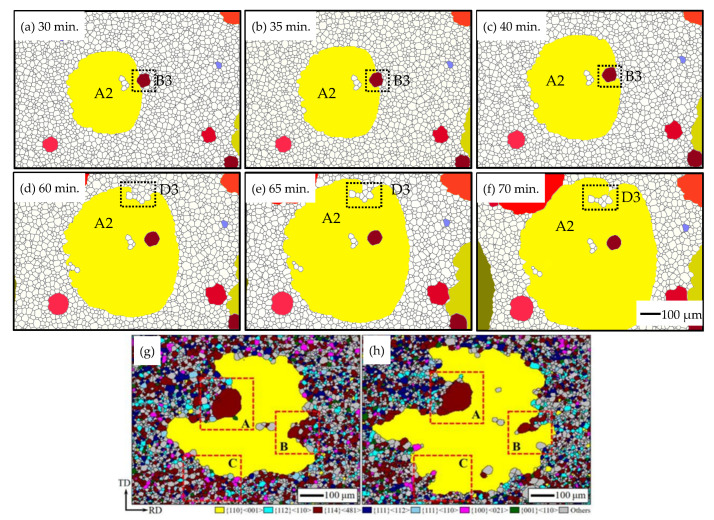
The formation process of island grains in: (**a**–**f**) simulated results, (**g**,**h**) experimental observations [49]. A2 is the isolated island grain, D3 is the serial island grain in the simulated results. A is the isolated island grain, B and C are the serial island grains in the experimental observations.

A detailed observation to capture the formation process of island grains is shown in Figure 15. The simulated results and experimental observations are consistent in finding two types of island grains [49]. As it shows that Goss grains could wrap around these grains and cause island grains to appear. It can be seen clearly that the island grain-B3 formed since A2 consumed its surrounding grains. Island grain-B3 also shrank a little, and thus it can also be consumed by grain A2 given enough time. As to the island grain-D4, there are several particles left at the GBs between the Goss grain A2 and the serial grains. It is difficult for this kind of serial island grain to be consumed by Goss grains unless the particles dissolve by means such as changing the temperature.

**Figure 16:**
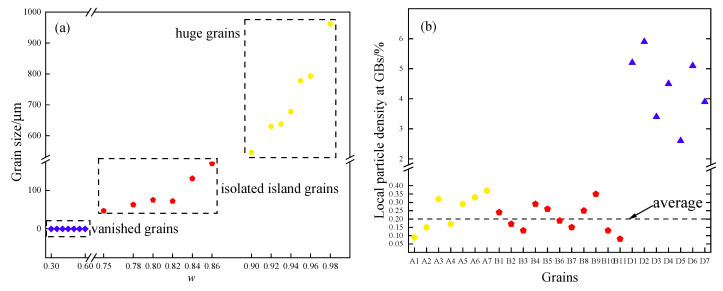
Quantitative analysis of the features of island grains at 140 min: (**a**) the relationship between *ω* and grain size; (**b**) local particle density at GBs, the blue triangles represent the local particle density at GB around serial island grains D1~D7.

A quantitative analysis was performed for the characterization of the two types of island grains, as shown in Figure 16. Figure 15a indicates the relationship between ω and grain size at 140 min annealing. It is clear that the grain size mainly depends on the GB dissolvability of particles, namely, the grains with a ω of 0.98~0.90 developed into huge grains, the isolated island grains mainly grew out of the grains with a ω of 0.86~0.75, and the rest grains with ω not higher than 0.75 were consumed during the annealing process. Figure 16b shows the local particle density at GBs, calculated by the ratio of the particle area to the GB area, around the GB of the serial island grains in Figure 14e. The particle density around the serial island grains is much higher than the average value of the whole simulated microstructure, and thus it can be known that the local particle density at GBs is the main factor for the formation of serial island grains.

Therefore, the isolated case shows the island grains are derived from some larger-sized grains (but much smaller than the huge abnormally grown grains such as A1~A7) which are not easily consumed away and surrounded by the abnormally grown grains; the serial case is due to a high-level undissolved particle in some regions, which leads to the pinning of GBs and protects the surrounding small grains. Thus, it can be summed up that the mechanism mainly relies on the following reasons: (1) A proper GB dissolvability of particles (ω = 0.86~0.75 in this simulation) causes the isolated island grains; (2) A higher local particle density at GBs contributes to the formation of the serial island grains.

## 4. Conclusions

Above all, a phase-field model coupled with the pinning effect caused by second-phase particles has been revised for the simulation of the microstructure evolution in Fe-3%Si steels. Based on Zener pinning theory, the particle content and dissolvability of particles at Goss GBs are controlled quantitatively through various threshold values. The following results are found:

1, The effect of second-phase particles on abnormal growth of Goss grains during the annealing process in Fe-3%Si steels has been studied by a revised phase-field model. Coupled with the pinning effect item in the model, the particle content and the dissolvability of Goss GBs to particles are controlled based on threshold values according to the Zener pinning theory. The simulated data agree well with the experimental results according to quantitative grain size analysis, which means the model is appropriate and the parameters are reasonable.

2, The influence of particle content and Goss GB dissolvability on the microstructure characteristics during the coarsening process is clarified. It is found that the abnormal growth level increases firstly and then decreases with an increasing particle content under a stable particle dissolvability of Goss GBs, whereas the abnormal growth level increases with a superior particle dissolvability under a constant particle content, which means the appearance of abnormal growth relies on the combination of the particle content and Goss GBs dissolvability. Meanwhile, the grain size advantage promotes further abnormal growth once the Goss grains undergo rapid growth.

3, Under a fixed particle content and Goss GBs with various particle dissolvabilities, two types of island grains can be found according to the simulated results, which consist of experimental observations. The mechanism mainly relies on the following reasons: (1) A proper GB dissolvability of particles causes the isolated island grains; (2) A higher local particle density at GBs contributes to the formation of serial island grains.

## Data Availability

The data presented in this study are available on request from the corresponding author.
